# Endodontic infection control practices among Pakistani general dental practitioners: A national cross-sectional questionnaire survey

**DOI:** 10.1016/j.jtumed.2023.05.014

**Published:** 2023-05-29

**Authors:** Muhammad Q. Javed, Mansoor Khan, Kiran I. Khan, Nawaf Almutairi

**Affiliations:** a Deparment of Conservative Dental Sciences, College of Dentistry, Qassim University, Buraidah, 52571, Qassim, Saudi Arabia; b Department of Operative Dentistry, Foundation University College of Dentistry, Foundation University, Rawalpindi, Pakistan; c Department of Operative Dentistry, Frontier Medical and Dental College, Abbottabad, KPK, Pakistan

**Keywords:** دراسات مقطعية, أطباء الأسنان, التطهير, علاج جذور الأسنان, مكافحة العدوى, استبانة, Cross-sectional studies, Dentists, Disinfection, Endodontics, Infection control, Questionnaire

## Abstract

**Objective:**

This study was aimed at evaluating the self-reported endodontic infection control practices of general dental practitioners in Pakistan.

**Methods:**

An e-questionnaire was sent to 619 general dental practitioners in several WhatsApp groups. Sixteen questions associated with various infection control measures recommended by the ESE were asked, including the use of various isolation methods/rubber dams, the selection of canal irrigants and anti-bacterial solutions, and practices regarding hand hygiene and use of examination gloves. The e-questionnaire also included questions associated with demographics. Data analysis was conducted in SPSS-24. Descriptive statistics were documented as percentages and frequencies.

**Results:**

Of 619 GDPs, 350 responded (56.5% response rate), of whom 43.7% worked in private dental practices. The majority were women (64%), had graduated after 2010 (81.1%), and were 24–34 years of age (78.9%). A total of 72.3% of GDPs used cotton rolls, and 17.4% routinely used rubber dams for endodontic isolation; 89% did not disinfect the operative field; 80% reported using different concentrations of NaOCl during root canal instrumentation; and 0.9% reported not using any irrigant during endodontic procedures. A total of 61.7% reported always using intra-canal medication during multi visit endodontic treatment, among whom 82.5% reported using Ca(OH)2. Finally, 100% of respondents reported using gloves during endodontic treatment.

**Conclusion:**

The results indicated that the GDPs tended to follow some of the endodontics quality standards recommended by the ESE, but the overall implementation of all guidelines requires improvement.

## المخلص

**أهداف البحث:**

كان الهدف من هذه الدراسة هو تقييم ممارسات مكافحة العدوى لممارسي طب الأسنان العامين في باكستان، كما أبلغوا ذاتيا من قبلهم.

**طرق البحث:**

تمت دعوة ما مجموعه 619 ممارسا عاما لطب الأسنان للمشاركة في هذه الدراسة عبر استبيان إلكتروني تم توزيعه في مجموعات واتساب المختلفة. يتكون الاستبيان من ستة عشر سؤالا تتعلق بتدابير مكافحة العدوى المختلفة التي أوصت بها الجمعية الأوروبية لطب لب الأسنان. تضمنت الأسئلة موضوعات مثل استخدام السد المطاطي أو طرق العزل الأخرى، واختيار أجهزة ري القناة والحلول المضادة للبكتيريا، والممارسات المتعلقة بنظافة اليدين واستخدام قفازات الفحص. كما تم جمع المعلومات الديموغرافية. تم تحليل البيانات باستخدام الحزمة الإحصائية للعلوم الاجتماعية، وتم الإبلاغ عن الإحصاء الوصفي كنسب مئوية وترددات.

**النتائج:**

من بين 619 ممارسا عاما لطب الأسنان، استجاب 350، مما أدى إلى معدل استجابة قدره 56.5٪. عمل حوالي 43.7٪ من ممارسي طب الأسنان العام في أجهزة طب الأسنان الخاصة. كانت غالبية المستجيبين من الإناث (64٪)، وتخرجن بعد عام 2010 (81.1٪)، وتنتمي إلى الفئة العمرية من 24 إلى 34 عاما (78.9٪). استخدم حوالي 72.3٪ من ممارسي طب الأسنان العام لفائف القطن، واستخدم 17.4٪ فقط السد المطاطي بشكل روتيني لعزل لب الأسنان. 89٪ لم يقوموا بتطهير مكان الجراحة. أفاد حوالي 80٪ من ممارسي طب الأسنان العام باستخدام تركيزات مختلفة من ناضور الصوديوم الهايبوكلورايت أثناء استخدام أجهزة قناة الجذر، بينما أفاد 0.9٪ أنهم لا يستخدمون أي جهاز ري أثناء إجراءات علاج لب الأسنان. أفاد 61.7٪ من ممارسي طب الأسنان العام أنهم يستخدمون دائما الأدوية داخل القناة أثناء العلاج اللبي متعدد الزيارات، وأعلن 82.5٪ من هؤلاء أنهم يستخدمون كالسيوم هيدروكسيد. أفاد جميع المستجيبين باستخدام قفازات اليد أثناء العلاج اللبي.

**الاستنتاجات:**

أشارت النتائج إلى أن ممارسي طب الأسنان العام كانوا يميلون إلى اتباع معايير جودة معينة في علاج جذور الأسنان على النحو الموصى به من قبل الجمعية الأوروبية لطب لب الأسنان، ولكن التطبيق العام لجميع المبادئ التوجيهية لم يكن مرضيا تماما ويتطلب التحسين.

## Introduction

The preservation of healthy pulp, maintenance of pulp vitality, minimally invasive therapeutic interventions, and prevention of apical periodontitis are the fundamental elements of contemporary endodontics.^1^ Endodontics is a broad field that encompasses therapies such as vital pulp therapy, pulpectomy, root canal treatment, non-surgical and surgical retreatments, non-vital tooth bleaching, and management of teeth that have undergone dentoalveolar trauma. Endodontic infection is a sequela of microbial invasion of the pulp. When infection of the root canal spreads to the periapical tissues, apical periodontitis develops. The aim of endodontic intervention is to eliminate bacteria and other microorganisms from the root canals and seal the canals to prevent re-infection; this aim is achieved by endodontic treatment (ET), whose success is determined by adequate healing of apical tissues.^2^, ^3^, ^4^

Strong evidence indicates that endodontic infections are not always attributable to the bacterial flora in the patient's oral cavity, and some sources of infection may be exogenous. Therefore, poor infection control and cross-contamination during ET can result in secondary endodontic infection.^5^ The most common cause of treatment failure is the presence or persistence of bacteria within the canals. According to the literature, complete eradication of microorganisms cannot be achieved by mechanical preparation and chemical disinfection of canals. However, bacteria can be maintained below a critical level by following strict aseptic protocols and improved technical standards, thereby increasing the success rate of ET.^6^ The European Society of Endodontology (ESE) has proposed treatment guidelines to prevent iatrogenic infections. These guidelines recommend meticulous hand hygiene, use of gloves, sterilization of instruments, isolation with rubber dams (RDs), disinfection of the operative field, aseptic technique, irrigation, and use of intracanal medications (ICMs).^2^ Using an RD is considered mandatory for the isolation of teeth during ET. Teeth treated endodontically without RD isolation are indicated for retreatment.^7^ Unfortunately, numerous studies have reported that most clinicians do not adhere to these guidelines.^8^, ^9^, ^10^, ^11^ Table 1 highlights the strategies that clinicians should use for elimination of endodontic infection and prevention of re-infection.^12^^,^^13^

**Table 1:** Strategies that clinicians should adopt to eliminate endodontic infection and prevent re-infection.

Operative strategies	Advantages
Isolation; use of rubber dam	Prevents contamination of the tooth under treatment with saliva
Operative field disinfection	Prevents entry of bacteria from the oral cavity into the root canal system by decontamination of the tooth surface
Seal between tooth and dental dam	Prevents salivary cross-contamination of the root canals and restorations
Sodium hypochlorite irrigation	Decreases bacteria from the areas that can not be accessed with endodontic instruments, and dissolves the organic components
EDTA irrigation	Dissolves the inorganic components and removes the smear layer
Final rinse with sodium hypochlorite	Disinfects dentinal tubules exposed during preparation and disinfection
Chemical disinfection of root canals	Decreases the chance of reinfection of root canals and increases treatment success
Discarding gloves and donning new gloves before obturation	Minimizes cross-infection from environmental sources
Intra-canal medication	Prevents re-infection and stunts bacterial growth, keeping bacterial load to a minimum between appointments
Alcohol-based hand disinfectant	Causes microorganisms to lose their protective coatings and become non-functional.
Clinic-specific routines for endodontic infection control	Ensures that dentists follow infection control guidelines and deliver a high standard of care to their patients

Infection control is a major factor affecting prognosis. To provide a high standard of care to patients, cross-contamination must be prevented at every step. The World Health Organization recommends hand hygiene as an effective and economical means of infection prevention.^14^ However, studies have reported that many healthcare personnel, even when being observed, do not abide by these hand hygiene recommendations.^15^ Root canal treatments performed by specialists have higher success rates than those performed by general dental practitioners (GDPs), possibly because specialists may have better clinical skills, infection control, and use of aseptic techniques.^9^^,^^10^ By placing high-quality root canal fillings, using aseptic techniques, and adhering to the infection control guidelines recommended by the ESE, oral health and ET outcomes can be greatly enhanced. Given the importance of infection control measures during ET and the lack of data on the endodontic infection control routines followed by Pakistani GDPs, this study was aimed at determining which endodontic infection prevention and control routines are followed by GDPs in Pakistan. Hence, we evaluated the self-reported endodontic infection control routines (EICRs) among GDPs in Pakistan. The findings of this study should enable self-evaluation of endodontic infection control routines by already practicing GDPs and indicate key areas of the undergraduate endodontic curriculum that should be modified to better instill infection control routines in future dentists.^16^^,^^17^ The study also highlights areas in which robust policy-making is required by the appropriate regulatory bodies for implementation of EICRs in Pakistan.^18^

## Materials and Methods

An e-questionnaire-based cross-sectional survey was performed on GDPs in Pakistan from August 2022 to October 2022. A sample size of 345 was calculated with the raosoft sample size calculator,^19^ on the basis of the total of 29,000 registered GDPs in Pakistan,^20^ a confidence level of 95%, and a margin of error of 5.25%. A non-probability purposive sampling technique was used.

This survey used a questionnaire from a study conducted by Malmberg and colleagues.^21^ The questionnaire comprised 16 questions associated with various infection control measures recommended by the ESE for implementation during ET, including the use of different isolation methods/RDs, the selection of canal irrigants and anti-bacterial solutions, and practices regarding hand hygiene and the use of examination gloves. The e-questionnaire also included questions associated with demographics.

The questionnaire, along with information regarding the survey, was sent to 619 GDPs in several WhatsApp groups. The GDPs were assured anonymity and informed that their participation was voluntary. Subsequently, two reminders at 2-week intervals were sent to the WhatsApp groups. The submission of filled questionnaires by GDPs was considered to imply consent. All information obtained was kept confidential. The GDPs submitting filled questionnaires were included in the study, whereas the non-responding GDPs were excluded from the study.

The data analysis was conducted in SPSS-24 (SPSS Inc., Chicago, IL). The descriptive statistics were documented as percentages and frequencies.

## Results

Of the 619 GDPs who were approached, 350 responded (response rate of 56.5%). All questions were answered by 100% of the respondents. Most GDPs (43.7%) worked in private dental practices, were women (64%), had graduated after 2010 (81.1%), were 24–34 years of age (78.9%) (Table 2), and had a median birth year of 1992. Most of the GDPs reported using cotton rolls (72.3%) during endodontic procedures, and only 17.4% reported routine use of RDs for ET. Almost 41% of the GDPs who used RDs during endodontic procedures reported using dental floss to achieve a seal at the RD-tooth interface. Most GDPs (89%) did not perform disinfection of the operative field; 80% of GDPs reported using different concentrations of NaOCl during root canal instrumentation; 0.9% reported not using any irrigant during endodontic procedures; 3.1% reported using NaOCl at high concentrations; 71.1% reported using additional irrigants during single visit endodontic procedures; and none of the GDPs reported using 5% IKI. Among the 61.7% of GDPs who reported always using ICMs during multi visit ET, 82.5% reported using Ca(OH)_2_ (Table 3).

**Table 2:** Demographics of general dental practitioners.

Age group (years)	n (%)	Year of graduation	n (%)	Gender	n (%)	Workplace hospital/clinics	n (%)
24–34	276 (78.9)	>2010	284 (81.1)	Female	224 (64)	Government	79 (22.6)
35–44	56 (16)	2001–2010	50 (14.3)	Male	123 (35.1)	Private	153 (43.7)
45–54	13 (3.7)	1991–2000	11 (3.1)	Prefer not to say	3 (0.9)	University	116 (33.1)
55–64	5 (1.4)	1981–1990	5 (1.4)			Others	2 (0.6)

**Table 3:** Disinfection and isolation practices during endodontic procedures.

**Question items**	**Options n (%)**					
**Tooth isolation with:**	Nothing		Cotton roll		Rubber dam	
	36 (10.3)		253 (72.3)		61 (17.4)	
**Rubber dam sealing**	No	Always	Sometimes	Do not use rubber dam		
	9 (2.6)	25 (7.1)	27 (7.7)	289 (82.6)		
**Sealing methods**	Dental floss	Do not seal	Oraseal	GIC/comp∗	ZOE/IRM∗	Do not use rubber dam
	30 (8.6)	10 (2.9)	2 (0.6)	1 (0.3)	18 (5.1)	289 (82.6)
**Operative field disinfection**	Do not disinfect	3% H_2_O_2_	30% H_2_O_2_	0.5–1% NaOCl	5–10% iodine tincture	0.5% CHX in 70% alcohol
	312(89%)	7 (2%)	0(0%)	28 (8%)	3 (0.9)	0(0%)
**Irrigant during instrumentation**	0.5% NaOCl	1% NaOCl	0.5% CHX	Saline	Nothing	5.25% NaOCl
	177 (50.6)	92 (26.3)	9 (2.6)	58 (16.6)	3 (0.9)	11 (3.1)
**Additional irrigant use in single visit endodontic Rx**	No	Yes, always	Yes, when treating pulpitis		Yes, when treating apical periodontitis	
	99 (28.3)	190 (54.3)	36 (10.3)		25 (7.1)	
**Additional irrigant type use in single visit endodontic Rx**	None	0.5% CHX	5% IKI	15–17% EDTA	Others (mostly 2% CHX)	
	99 (28.3)	86 (24.6)	0 (0)	144 (41.1)	21 (6)	
**Inter-appointment intra-canal dressing placement**	None	Yes, always	Yes, when patient has pain	Yes, when treating pulpitis	Yes, when treating apical periodontitis	
	7(2)	216(61.7)	47 (13.4)	21 (6)	59(16.9)	
**Intra-canal dressing type**	Nothing	0.5%CXN	5%IKI	Ca(OH)_2_ paste	TAP	Cresophene
	7 (2)	12 (3.4)	15 (4.3)	290 (82.9)	6 (1.7)	20 (5.7)

Although 100% of the participants reported using hand gloves during ET, only 64.7% reported using hand disinfectants. Of the 66.1% of participants who indicated that their clinics have specific antiseptic routines for endodontics, 16% reported that they do not follow these routines (Table 4).

**Table 4:** Use of gloves and hand disinfectant during endodontic treatment.

Gloves use timing	n (%)	Use of hand disinfectant	n (%)	Clinic's infection control routine	n (%)
**During entire course of the treatment**	333 (95.1)	**Yes**	229 (64.7)	**No**	66 (18.9)
**Before operative field has been disinfected**	2 (0.6)	**No**	123 (35.1)	**Yes, and I follow them**	193 (55.1)
**After operative field has been disinfected**	15 (4.3)			**Yes, but I follow my own routine**	56 (16)
**Do not use gloves**	0 (0)			**I do not know**	35 (10)

## Discussion

The response rate in the current study was high (56.5%) with respect to those in similar studies that disseminated the study questionnaires through postal mail.^22^, ^23^, ^24^, ^25^ The use of WhatsApp for delivering the e-questionnaire and the subsequent follow up ensured an adequate response from GDPs. This finding is indicative of a declining preference among members of the dental community to respond to surveys delivered through traditional postal services, as well as the effectiveness of social media platforms to achieve high reach and response rates, possibly because of ease of use and the rapidity of filling and submitting questionnaires electronically.

One critical factor ensuring the maintenance of an aseptic environment during ET is dental RD use. The ESE quality guidelines for ET, American Association of Endodontics guide to clinical endodontics, and British Endodontic Society guide to good endodontic practice refer to RD use as an “essential” and “integral” part of ET and a quality standard.^26^, ^27^, ^28^ Most GDPs enrolled in this study reported using cotton rolls and suction for isolation, whereas 17.4% of the GDPs applied RDs during ET. The percentage of GDPs using RDs in the current study was low with respect to those reported in studies performed in the USA, Germany, and Switzerland, where the percentage of RD use ranges from 56% to 63%.^22^^,^^23^^,^^28^ In contrast, our results are comparable to those of Dogra et al. and Gupta et al. in India, where 21% and 27% dentists, respectively, apply RDs during ET.^29^^,^^30^ Likewise, low RD use has been observed in Iran, KSA, Syria, and Turkey, with use rates ranging between 2% and 16.5%.^31^, ^32^, ^33^, ^34^ A study in Denmark has highlighted that recent graduates are more inclined to use RDs (29%) than more experienced clinicians (4%).^24^ The application of RDs is mandatory in dental colleges across Pakistan at the undergraduate level. However, increased chairside time/cost, limited patient awareness/acceptance, and the lack of robust regulation of RD application may be several reasons why most of the dentists in this study did not continue to apply RDs after graduation.

Teeth with deep class 2 carious lesions or extensive structural loss might require pre-endodontic build-up, use of a caulking agent, or RD inversion with floss ties to provide an additional seal. Of the 17.4% GDPs reporting applying RDs in the survey, 8.6% reported using floss, 5.1% reported using IRM, 0.3% reported using pre-endodontic build up with GIC or composite, and 0.6% reported using Oraseal to achieve a seal where required. Moreover, 2.9% of the RD users reported that they did not use any additional sealing strategy. Malmberg et al. have reported use rates of Oraseal and liquid RDs by 40.8% and 27.6% of GDPs, respectively. In addition, 31.6% of dentists reported using dental floss for enhancing the seal around the teeth isolated by RD in that study, and 97% participants reported using RDs during endodontic procedures. The results indicate an increased emphasis on RD isolation during ETs among Swedish and Norwegian dentists, possibly because of affordability and increased patient awareness.^21^ The low rate of use of a dedicated sealing material such as Oraseal in our study might be attributable to the high cost and relative lack of availability of this material in Pakistan.

Operative field disinfection has not been extensively addressed in endodontic surveys. In the present study, fewer than 11% of the GDPs indicated that they disinfect the RD and tooth to be treated before access cavity preparation. The most commonly used disinfectant was 1% NaOCl. None of the dentists reported using the recommended concentration of 30% hydrogen peroxide,^35^ possibly because of the already low RD use and the scarce commercial availability of 30% H_2_O_2_ in Pakistan. In contrast, Malmberg has reported that 30% H_2_O_2_ for operative field disinfection was used by 53.6% of Swedish and Norwegian GDP survey respondents, and the most commonly used disinfecting solution was a combination of 0.5% CHX and 70% alcohol.^21^ The findings of that study have also indicated a reluctance to use higher concentrations of H_2_O_2_, which is caustic if it comes in contact with oral tissues and is also expensive. The effectiveness of alternative disinfectants, such as 1% NaOCl and a 0.5% CHX and 70% alcohol mixture, for disinfecting the operative field must be validated in future studies.

RCS disinfection is performed during cleaning and shaping by mechanical debridement with ET files, and chemical disinfection is performed with endodontic irrigants (EIs). Ideally, EIs should be antibacterial, have organic tissue dissolution ability, act as a lubricant, and be able to remove the smear layer to achieve better penetration of the irrigants into the dentinal tubules.^36^^,^^37^ No single EI has all the aforementioned properties. Hence, a combination of EIs is required to achieve thorough RCS disinfection. The most effective EI is NaOCl which has both antibacterial and organic tissue dissolution properties. However, the inability of NaOCl to remove the smear layer requires the use of an additional EI with a chelating agent, such as EDTA, to enhance the penetration NaOCl into the dentinal tubules. In this study, 80% of the participants reported using NaOCl as the irrigant during canal instrumentation, and 0.5% was the most preferred concentration. The preference for lower concentrations might have been associated with the low rate of RD use: 16.6% reported using saline as the sole irrigant, whereas 0.9% reported using no irrigant at all. The rate of NaOCl use reported among Swiss dentists is similar to that found in our study: 90% of Swiss dentists have reported using NaOCl for canal irrigation, and 0.5% is the preferred concentration.^28^ However, that study included both GDPs and endodontic specialists. More than 54% of the respondents reported using an additional EI during ET. EDTA was the most commonly used additional EI (41.1%). A high percentage (67.7%) of Swiss dentists have been reported to use smear layer removal treatment.^28^ Gupta et al. have reported NaOCl use among 33% of the dentists in India, whereas 36% use saline, and 14% use hydrogen peroxide as an ICM. Most practitioners in the above-mentioned study were either recent graduates or clinicians who had been practicing for fewer than 10 years. Thus, a slow rate of uptake of newer technologies and trends by Indian dentists has been reported.^30^ However, that study was conducted in 2011, and increases in use rates are expected to have occurred since then. A substantial number of dentists in our study reported using NaOCl for disinfection and EDTA for smear layer removal, possibly because most dentists who responded were recent graduates, and dental schools in Pakistan place special emphasis on the use of NaOCl for canal disinfection and EDTA for smear layer removal.

Additionally, the use of ICMs has been proposed to supplement RCS disinfection and promote periapical healing, particularly in necrotic cases.^38^^,^^39^ Calcium hydroxide (CH) is the most commonly used ICM, owing to its anti-bacterial activity and ability to promote periapical healing by providing an alkaline environment.^40^ We found that 82.9% of the current survey respondents reported using CH as an ICM. This result corresponded to the CH use rates documented worldwide.^22^^,^^28^^,^^30^^,^^31^^,^^33^^,^^34^ Approximately 5.7% of the participants reported using cresophene as an ICM. Cresophene contains camphor and parachlorophenol as the main anti-bacterial agents, both of which are toxic to human cells.^41^^,^^42^ Moreover, cresophene has questionable anti-bacterial activity.^43^^,^^44^ Therefore, it is no longer recommended for use in endodontics, because of safety concerns. Continued use of cresophene was observed among experienced dentists in this study, thus indicating a lack of knowledge of current trends regarding ICMs in endodontics.

All GDPs who participated in the study wore gloves during ET: 95% reported using a single pair of gloves during the entire procedure, two participants indicated using new gloves before disinfecting the operative field, and 15 participants reported donning new gloves after disinfecting the operative field. The ESE quality guidelines suggest changing gloves at least before the start of obturation, because the exposure of gloves to the oral environment at the time of RD placement as well as to endodontic pathogens during the course of the treatment contaminates the gloves and may compromise the sterility of the operative field.^45^^,^^46^ Approximately 64% of the dentists in this study indicated that they disinfect their hands with alcohol-based disinfectant before donning gloves. However, whether the dentists who change gloves during treatment also apply disinfectant between glove changes was unclear. We believe that the use of sterile surgical gloves after operative field disinfection and surgical hand scrubbing should be considered essential by the dental community, particularly during surgical retreatment procedures, to provide robust cross infection control.

### Strengths and limitations

This is the first study focusing on the endodontic infection control practices of GDPs working in Pakistan. The study has several limitations. First, the participants were not asked about the use of several technologies that are essential for proper disinfection of the RCS, such as the method used for activation of irrigants and the use of microscopes. Second, most responding GDPs were recent graduates and thus their responses might not represent common practice among GDPs in Pakistan. Third, the study evaluated self-reported routines of GDPs, which may not be truly representative of GDPs’ actual behavior.

### Future recommendations

Future studies with larger sample sizes and inclusion of more experienced GDPs are recommended. Moreover, research should be conducted to explore the correlation between GDPs’ self-reported EICRs and actual EICRs, with the inclusion of questions associated with contemporary technologies.

## Conclusions

The results indicated that the GDPs tended to follow some of the endodontics quality standards recommended by the ESE, but the overall implementation of all guidelines requires improvement. These findings suggest a need for further training of undergraduate dental students in Pakistan and the incorporation of training associated with EICRs.^47^ Moreover, the introduction of mandatory endodontics-associated CME activities by the regulatory body for license reaccreditation would enhance GDPs’ awareness and implementation of EICRs in clinics. These steps may also aid in improving the overall quality of endodontic practice among GDPs in Pakistan.

## Source of funding

This research did not receive any specific grant from funding agencies in the public, commercial, or not for-profit sectors.

## Conflict of interest

The authors declare no conflicts of interest.

## Ethical approval

Study approval was acquired from the Institutional Review Board on March 15, 2020 (IIDC/IRC/2020/003/012) at the faculty of dentistry, Riphah International University, Islamabad, Pakistan.

## Authors contributions

MQJ: Study conception and design, data collection, analysis, table preparation, write-up, final editing/review of the manuscript, and supervision. MK and KK: Write-up. NA: Study conception and design, and final review of the manuscript. All authors have critically reviewed and approved the final draft and are responsible for the content and similarity index of the manuscript.
