# Supplementary material for: Endodontic infection control practices among Pakistani general dental practitioners: A national cross-sectional questionnaire survey
Source: J Taibah Univ Med Sci. 2023 May 29;18(6):1342–9. doi: 10.1016/j.jtumed.2023.05.014 (PMC10248868; doi:10.1016/j.jtumed.2023.05.014)
Supplement: Multimedia component 1 [file mmc1.docx]

| **S.no** | **Term** | **Operational definition** | **Reference** |
| --- | --- | --- | --- |
| 1 | Dental dam (rubber dam) | A small latex or non-latex dam used to isolate a tooth or teeth from the oral environment and to prevent migration of fluid and foreign objects into or out of the operative field; single or multiple holes punched through the barrier allow for placement around the tooth or allow teeth to be isolated. Dams provide a dry, visible, and clean operative field. | https://www.aae.org/specialty/clinical-resources/glossary-endodontic-terms/  file:///C:/Users/qasim/Downloads/Glossary%20Of%20Endodontic%20Terms%20-%20UPDATED%20MARCH%202020%20050720.pdf |
| 2 | Disinfection | A non-specific term implying the destruction of pathogenic bacteria, but not necessarily spores, usually by chemical agents. |  |
| 3 | Intracanal medication | A chemical agent sealed within the root canal system, used between appointments as an anodyne and or antimicrobial agent. |  |
| 4 | Irrigant | Liquids used for intracanal irrigation; examples include sodium hypochlorite, saline, chlorhexidine, hydrogen peroxide, and EDTA. |  |
| 5 | Infection control | The operator and dental nurse should wear gloves and use aseptic technique. All instruments used within the oral cavity should be sterile, or should be decontaminated and sterilized or disinfected if sterilization is not possible. The tooth should be isolated with a rubber dam. The tooth to be treated and the rubber dam should be disinfected before entering the pulp cavity. | Löst C. Quality guidelines for endodontic treatment: Consensus report of the European Society of Endodontology. Int Endod J. 2006;39(12):921–30. |
